# Drive-amplitude-modulation atomic force microscopy: From vacuum to liquids

**DOI:** 10.3762/bjnano.3.38

**Published:** 2012-04-18

**Authors:** Miriam Jaafar, David Martínez-Martín, Mariano Cuenca, John Melcher, Arvind Raman, Julio Gómez-Herrero

**Affiliations:** 1 Departamento de Física de la Materia Condensada, Universidad Autónoma de Madrid, E-28049 Madrid, Spain; 2 Servicios generales de apoyo a la investigación, Universidad Autónoma de Madrid, E-28049 Madrid, Spain; 3 Department of Engineering Mathematics, University of Bristol, Bristol BS8 1TR, United Kingdom; 4 Birck Nanotechnology Center and School of Mechanical Engineering, Purdue University, West Lafayette, IN 47904-2088, USA

**Keywords:** atomic force microscopy, control systems, dissipation, frequency modulation, noncontact

## Abstract

We introduce drive-amplitude-modulation atomic force microscopy as a dynamic mode with outstanding performance in all environments from vacuum to liquids. As with frequency modulation, the new mode follows a feedback scheme with two nested loops: The first keeps the cantilever oscillation amplitude constant by regulating the driving force, and the second uses the driving force as the feedback variable for topography. Additionally, a phase-locked loop can be used as a parallel feedback allowing separation of the conservative and nonconservative interactions. We describe the basis of this mode and present some examples of its performance in three different environments. Drive-amplutide modulation is a very stable, intuitive and easy to use mode that is free of the feedback instability associated with the noncontact-to-contact transition that occurs in the frequency-modulation mode.

## Introduction

**Figure 1:**
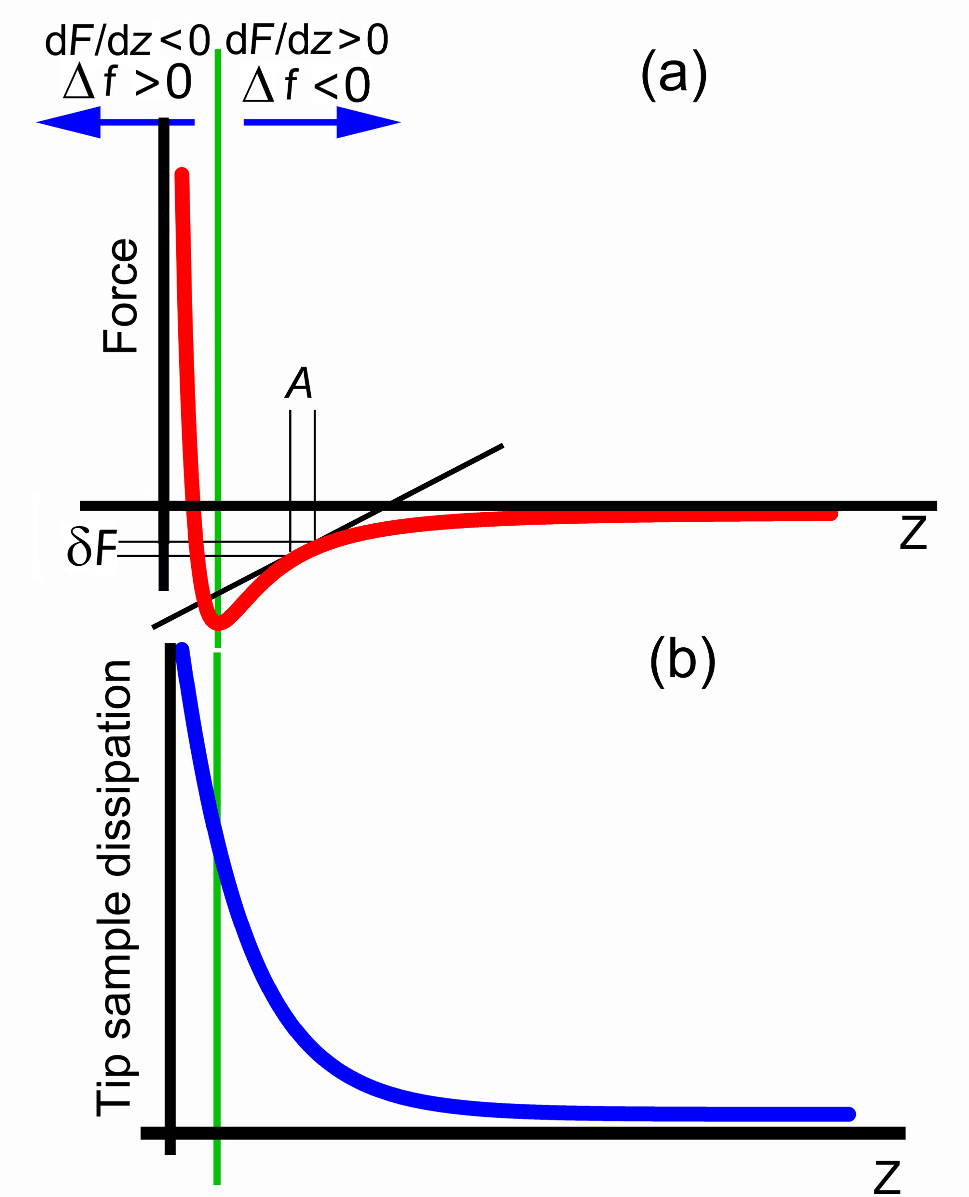
The interaction versus distance. (a) Conservative force versus distance interaction between an AFM tip and a surface. As the tip approaches the surface the interaction becomes first attractive and then repulsive. The frequency shift also varies from negative to positive. FM is only stable in one of the two branches. (b) In addition to the conservative interactions the tip also dissipates energy when interacting with the surface. The figure illustrates the monotonic tendency of this magnitude.

Dynamic atomic force microscopy (dAFM) [1–2] is a powerful yet versatile tool capable of operating in environments ranging from ultrahigh vacuum (UHV) to liquids [3–4], and imaging samples ranging from stiff inorganic materials [5] to soft biological matter [6], with nanoscale resolution. Amplitude-modulation AFM (AM-AFM) [7] and in particular its large-amplitude version, commonly known as tapping mode [8], is the most extended dAFM mode, but it has limitations: Its application to the vacuum environment is very difficult because of the long scanning times imposed by the high quality factor Q of the cantilevers in vacuum, which present a settling time given by τ*_cl_=* Q*/*(π*f*_0_). Frequency-modulation AFM (FM-AFM, also known as noncontact AFM) [9] is the classical alternative to AM allowing atomic resolution in UHV chambers [10] at higher scanning rates. FM-AFM has recently been extended to operate in other media with lower Q, with remarkable success [11]. However, FM-AFM has a well-known drawback: The transition from noncontact to contact causes an instability in the feedback control [12], which is particularly important for inhomogeneous surfaces in which, for example, the adhesion changes abruptly. The curve in Figure 1a represents a typical curve of the tip–sample force versus distance in a vacuum or air environment. The FM feedback maintains the frequency shift, which is closely related to the force gradient, to infer the topography of the sample [13]. Since the frequency shift changes its sign (Figure 1a), stable feedback is only possible on a branch of the force curve where it is monotonic. For the case of AM, the transition between the contact and noncontact regimes can introduce bistabilities [14–15] but, as a general rule, AM can operate with similar feedback conditions in both regimes. In liquid, the absence of significant van der Waals forces results in a monotonic interaction [4] and the feedback in both FM and AM is often perfectly stable. However biological samples, such as viruses, tend to contaminate the tip and introduce attractive interactions causing FM to become unstable. As we shall see, in these cases imaging biological samples with FM is impractical. In an attempt to overcome this control instability, we have developed the method presented herein. In addition to the conservative interactions depicted in Figure 1a, there exist nonconservative or dissipative forces, that subtract energy from the oscillation [16–17]. The dissipation generally grows monotonically [18] as the tip approaches the sample surface (Figure 1b). However, the precise dependence of the dissipation on the tip–sample distance depends on the detailed atomic configuration of the tip involved in the experiment [19].

In this work we present a new AFM scanning mode, which we have called “drive amplitude modulation” (DAM-AFM) [20] and which takes advantage of the aformentioned monotonicity of the dissipation to obtain stable images in all environments from vacuum to liquids. Moreover, DAM has a similar settling time to FM, and consequently the scanning time is also very similar. The paper begins by describing the basics features of DAM and comparing them with AM and FM, following by a discussion of some experimental results in vacuum and liquids.

## Results and Discussion

### The basis of DAM-AFM

**Figure 2:**
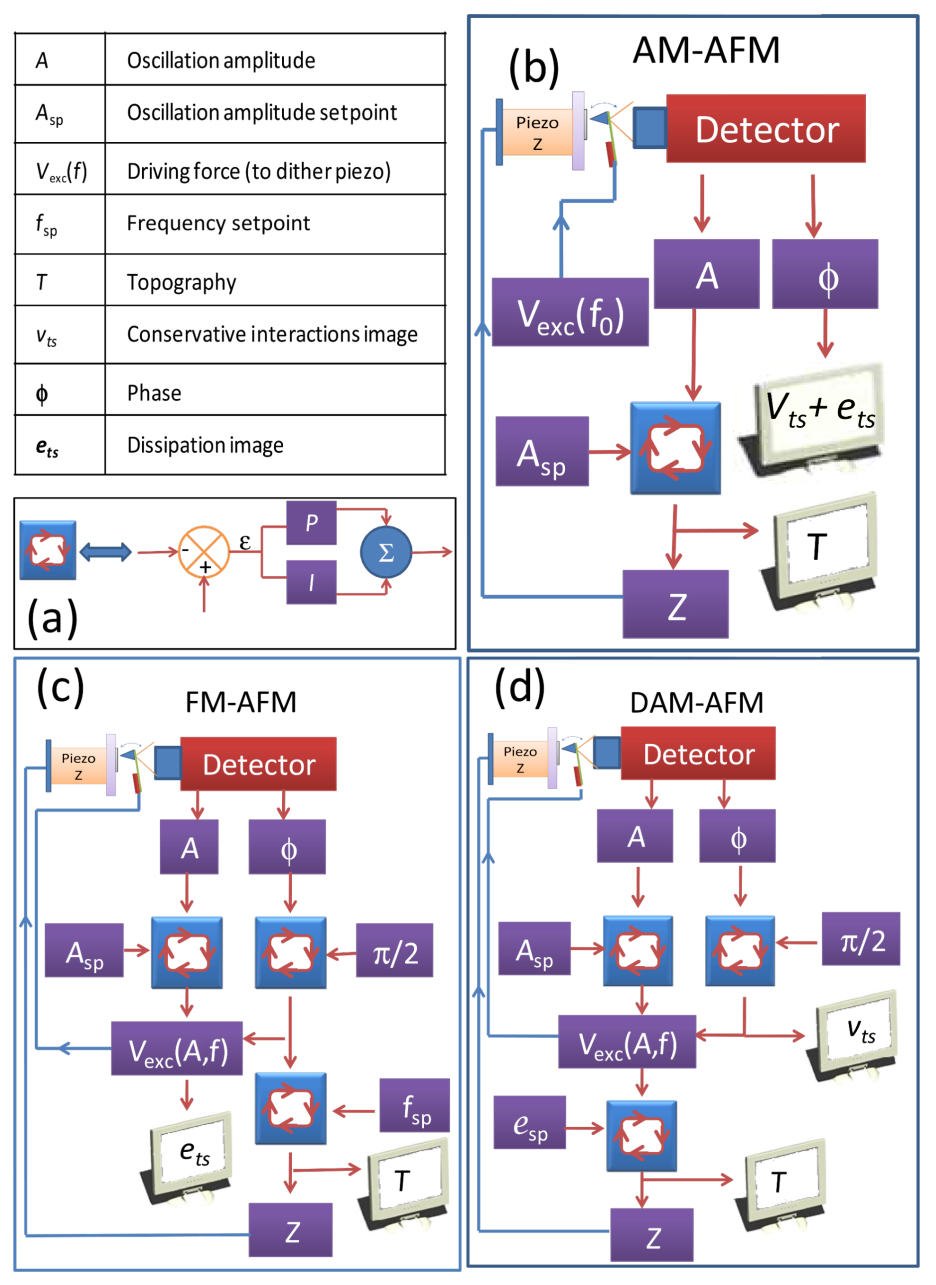
Feedback diagrams for different d-AFM modes. dAFM has three basic variables: The oscillation amplitude A, the phase 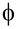 and the driving force *V_exc_*. (a) Expansion of the feedback icon used in the schemes. (b) Typical feedback scheme for AM. (c) FM feedback scheme. The short branch varies the driving force to keep the amplitude constant, hence producing a dissipation image (*e_ts_*). The other branch is a phase-lock loop, which keeps the system at resonance according to the tip–sample interaction. The regulated variable of the PLL, the frequency, is used as the controlled input for the topography feedback. (d) In DAM the short branch is a PLL, which produces a map of the conservative force (*v_ts_*). The long branch uses the amplitude as the process variable, and the regulated variable is the driving force, which is used as the controlled input for the topography feedback.

Figure 2 portrays the functional schemes for the three different AFM modes under consideration. The standard representation of a feedback loop and the corresponding icon used to simplify the different diagrams is shown in Figure 2a. For the case of AM (Figure 2b) a harmonic driving force with constant amplitude at (or near to) the free resonance frequency *f*_0_ of the cantilever is used. The oscillation amplitude *A* is the controlled input for the topography feedback, and the scanner position in the z-direction (perpendicular to the sample surface plane, and which is closely related with the tip–sample distance) is the regulated variable; the variation of the phase is recorded in the phase image, which is used as a spectroscopic image. In FM (Figure 2c) three feedback loops are used; two nested loops for the topography and one additional loop working in parallel to keep the oscillation amplitude constant by adjusting the amplitude of the driving force. A phase-locked loop (PLL) tracks the effective resonance frequency of the cantilever as it varies as a consequence of the tip–sample interaction. In FM, the position of the scanner in the z-direction is adjusted to keep the frequency shift constant and generates a topography image. This topography image is usually interpreted as a map of constant force gradient. The amplitude of the driving force, which is controlled in the parallel feedback loop, represents the dissipation. Figure 2d shows the functional scheme for DAM. As in FM, two nested feedback loops give the topography in DAM. The first loop adjusts the driving force in order to maintain the oscillation amplitude. The driving force needed to sustain this oscillation amplitude is related to the energy dissipated in the system. By adjustment of the position of the scanner in the z-direction the driving force is kept constant at the setpoint value. A PLL, which tracks the effect resonance frequency, can operate as parallel feedback loop in DAM. Topography images in DAM represent maps of constant dissipation. The frequency shift controlled by the PLL provides a spectroscopic image. We note that a PLL can also be implemented in AM. In this configuration the topography images in both AM and DAM have a similar meaning. Strictly speaking DAM can work with or without a PLL. In either case, the scanning speed in vacuum is comparable to that in FM. Nevertheless, while omission of the PLL simplifies the acquisition setup, the topography images, as in AM, reflect both conservative and nonconservative forces.

Notice that, as reflected in the schemes, in both FM and DAM the amplitude *A* and frequency *f* of the driving force


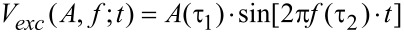


are modified by feedback loops that work with characteristic times τ_1_ and τ_2_ (not necessarily the same for frequency or amplitude) that depend on the details of the experimental setup but, as we will show, can be pushed well below the transient time of the free driven cantilever τ*_cl_*. What defines the difference between these two modes is which of the feedback loops working on this driving signal (amplitude for DAM or frequency for FM) is used as the process variable for the topography feedback.

All of the experiments described in this work have been carried out with Nanotec Electronica (http://www.nanotec.es) microscopes controlled with the SPM software package WSxM [21]. However, this mode can be easily implemented in other commercial systems. Nanosensors PPP-NCH and Olympus OMCL-RC type probes were used for the experiments in vacuum and in liquid, respectively. For the sake of completeness, in Supporting Information File 1 we also include images taken with other cantilever types. The stiffness values for each cantilever were obtained in an air environment by using Sader’s expression [22].

### In vacuum DAM-AFM

The experimental setup consists of a home-made high-vacuum chamber with a base pressure of 10^−6^ mbar, equipped with an AFM head. The vacuum is achieved by using a conventional combination of a dry mechanical pump plus a turbopump. In order to avoid vibrations from the turbopump affecting the measurements, the microscope head is suspended by three viton cords. The quality factor of the cantilevers saturates at pressures below 10^−3^ mbar, and hence the dynamics of the cantilevers are similar to what is typically observed in UHV chambers at room temperature (the values of the Q factor in UHV operation are commonly between 8000 and 25000). All the experiments were carried out at room temperature.

**Figure 3:**
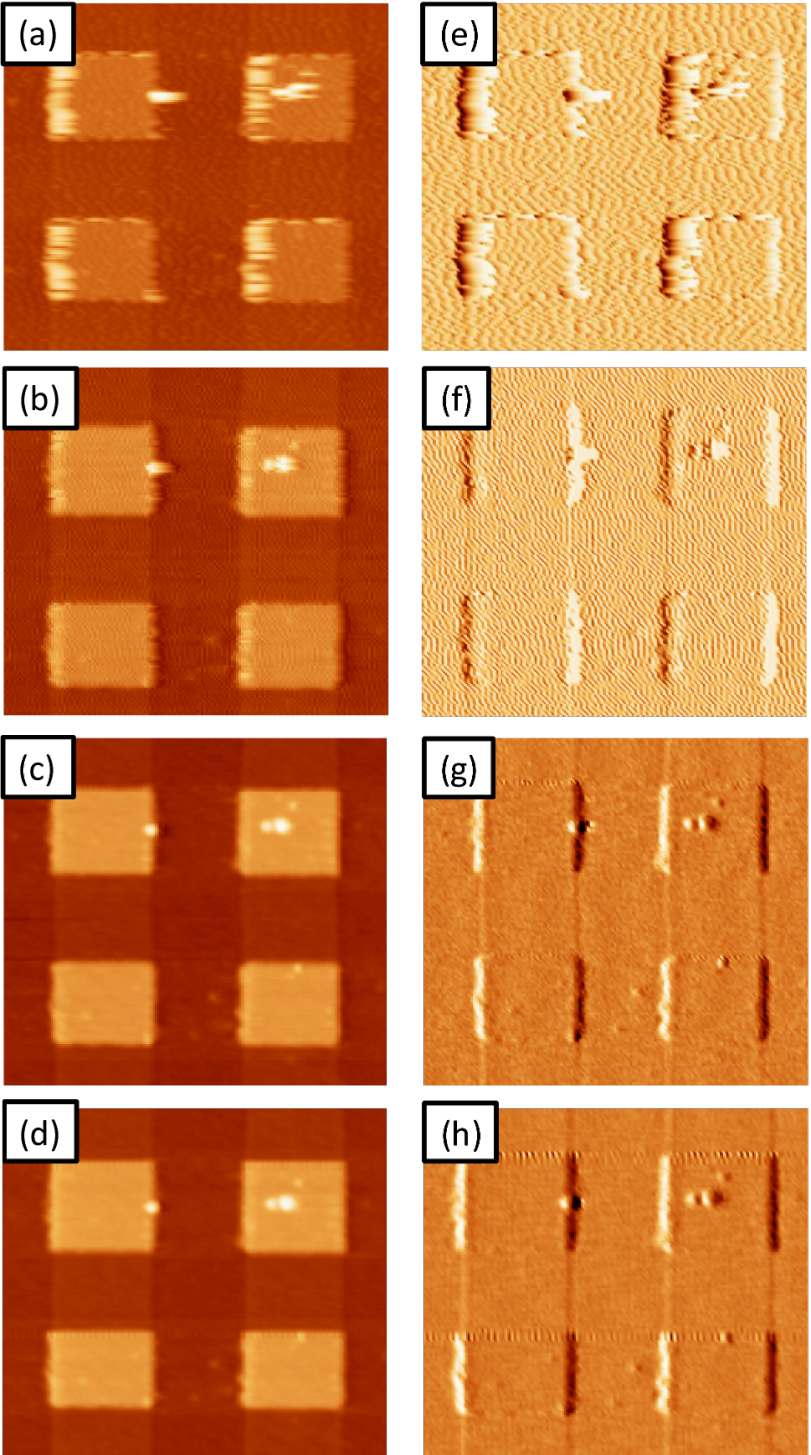
Testing the methods at high Q. Topography images of a calibration grid taken in vacuum in (a) AM (setpoint = 6.5 nm); (b) FM (setpoint = −50 Hz); (c) DAM in the attractive regime (setpoint = 1.2 pW; *V_exc_* = 0.49 V); and (d) DAM in the repulsive regime (setpoint= 4.5 pW; *V_exc_* = 0.77 V). (e–h) Corresponding error images: amplitude for AM, frequency shift for FM and dissipation for DAM. For all of the images: free amplitude *A* = 10 nm. *K* = 23 N/m, Q = 11800, line rate= 1.2 Hz, *f*_0_ = 225 kHz. The height of the motifs is 20 nm and the structural period is 3 μm.

Figure 3a–d portrays four topography images of a calibration grid taken in AM, FM and DAM acquired in both the attractive and repulsive regimes, respectively. Figure 3e–h shows the corresponding error signals: Amplitude, frequency shift, and dissipation for the two DAM cases, respectively. We have chosen this sample because its surface conditions are similar to those found in many samples of technological interest, and which in many cases are difficult to scan in vacuum by using a conventional mode. Scanning with DAM overcomes these difficulties. Figure 3a (AM) shows clear traces of instabilities as expected for AM images acquired at high Q for which the settling time is *τ_cl_* ≈ 17 ms, making this mode too slow for vacuum applications. In order to achieve higher scan rates the settling time can be reduced by increasing the tip–sample dissipation (diminishing the Q), which implies a large amplitude reduction and therefore higher applied forces during imaging. The frequency shift setpoint for Figure 3b (FM) is negative indicating that the topography image was taken in the attractive/noncontact regime (as is the usual case in FM). Imaging in FM at low amplitude was unstable because of the high adhesion observed on the surface: The interaction passes from being attractive to repulsive. To avoid this effect, we have to increase the feedback gain resulting in the appearance of high-frequency components in the error signal. In order to stabilize the system we used the *tip safe* option in the WSxM software, which prevents tip–sample crashes by withdrawing the tip when the oscillation amplitude of the cantilever drops below a given threshold. As usual we tried to optimize the scanning conditions for the chosen amplitude; nevertheless we could not reduce the high-frequency artifacts observed in the image. Figure 3c and Figure 3d (DAM) were acquired by using dissipation setpoints of 1.2 pW and 4.5 pW, respectively, with the PLL enabled, as calculated following the expression [23–24]

[1]
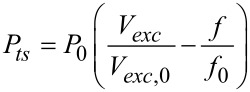


where *P*_0_ is the power dissipation caused by internal friction in the freely oscillating cantilever given by

[2]
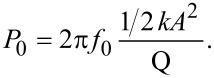


Stable imaging in DAM does not require *tip safe* or any other kind of precaution. Acquiring images in DAM is easy and direct. It is also possible to select the optimum cantilever oscillation amplitude for each experiment, ranging from less than 1 nm up to tens of nanometers at high scan speeds.

**Figure 4:**
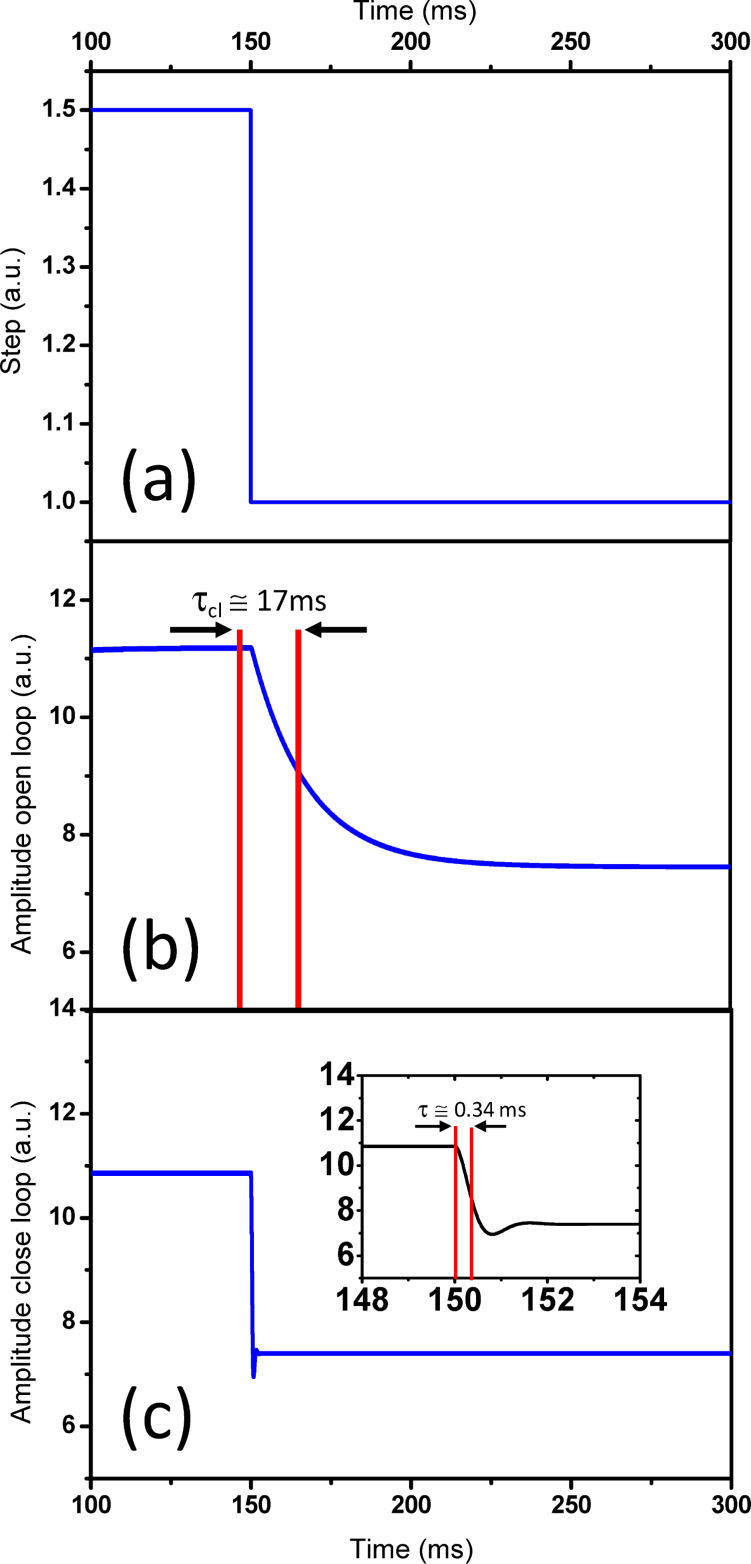
Response to a step perturbation under high Q. (a) Perturbation applied to the free cantilever. (b) Amplitude response for a free cantilever in the open-loop configuration. (c) Amplitude response for a free cantilever in the close-loop configuration. The inset shows a zoom in the step region, showing a characteristic time of 0.3 ms, which is much shorter that the one observed in the open-loop configuration. The MATLAB sequence diagram is shown in Supporting Information File 2.

It is known from control theory [25] that a feedback loop can modify the differential equation that describes the dynamic of a plant (in the present case, the plant is the cantilever). As a consequence, the new transient time can be reduced arbitrarily by changing the feedback gains. This is conveniently illustrated in Figure 4 (see a more detailed discussion in Supporting Information File 2). This figure portrays a MATLAB simulation in which a perturbation (Figure 4a) is applied to a free cantilever with Q = 15000. The response of the cantilever without any feedback shows the expected transient with a settling time of τ*_cl_* = Q/(π*f*_0_) (Figure 4b). Figure 4c displays the response of the cantilever with the amplitude and the frequency feedback loops enabled. Notice that the shape of the perturbation is a step function for both cases. However, for the open-loop case the perturbation is a sudden change in the amplitude of the driving force, whereas for the closed-loop configuration the perturbation is a sudden change in the amplitude setpoint. As shown in the charts, the response time in the second configuration is dramatically reduced with respect to the open-loop configuration.

**Figure 5:**
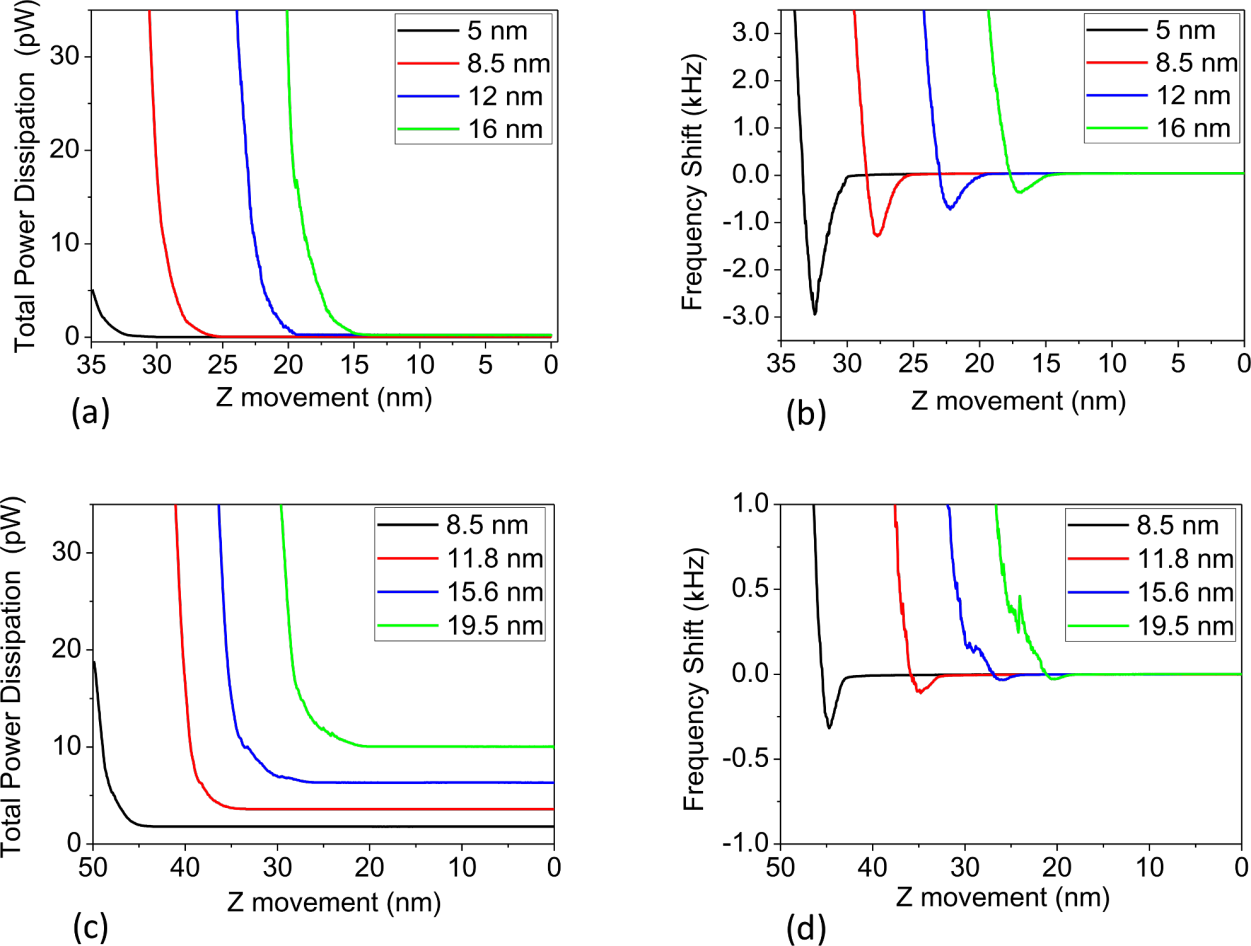
*In vacuum* total dissipation (a) and frequency shift (b) curves as a function of the z-scanner position for different amplitudes. (c) and (d) equivalent to the cases in (a) and (b) but *in air* (ambient conditions). The energy required to sustain the free oscillation in air is a factor of Q*_vacuum_*/Q*_air_* times the energy needed in vacuum. Cantilever parameters: *k =* 16.6 N/m, ω_0_ = 230.97 kHz, Q*_vacuum_ =* 23900*,* Q*_air_* = 468.

The second consideration, closely related to the previous one, is the energy balance. Assuming a free cantilever at resonance, the power that has to be provided to the cantilever to achieve a given amplitude is inversely proportional to Q (Equation 2). The implication is that keeping the cantilever at resonance in air requires *r*-times more power than in vacuum (being that *r =* Q*_vac_*/Q*_air_*). This *r* factor is about 20 for the cantilevers used in this work, but it can be much higher. Figure 5 shows the total dissipation and the frequency shift (simultaneously acquired) as a function of the z-scanner position for experiments, in both *vacuum* (a,b) and *air* (c,d). As expected, the power required to sustain the cantilever oscillation is much higher for the *in air* case than for the *in vacuum* case. In addition, the charts are experimental illustrations of the force and dissipation trends shown in Figure 1. The onset of both frequency shift and dissipation depends on the cantilever oscillation amplitude for obvious reasons: As the amplitude grows the tip finds the sample surface at a lower z-scanner position. When the tip approaches the surface it encounters a potential well that is the combination of the harmonic potential of the cantilever plus the surface potential. In order to maintain the oscillation we have to provide a total energy to the cantilever that is high enough that the tip is not trapped by the surface potential. Since the system is not conservative this total energy varies with time.

The energy dissipated by a cantilever over one period *in vacuum* is, as a consequence of the tip–sample interaction, on the order of 10^−20^ J (see, for instance, [26]). The energy required to force a cantilever to oscillate in vacuum with an amplitude of 10 nm is about the same as the energy loss per oscillation period. *In air* the energy required by the cantilever to maintain a stable free oscillation is 20 times higher, so the energy loss due to the tip–sample interaction is usually negligible. As a general rule, in order to enhance the sensitivity, the cantilever oscillation amplitude should be comparable to the selected interaction length [1–2]. Since in AM the energy pumped into the cantilever is fixed, the tip gets easily trapped in the sample potential and the image becomes unstable. This effect is particularly relevant in vacuum. In air and liquids the cantilever dissipation originated by the environment is much higher than the dissipation due to tip–sample interaction. Thus, the energy required by the cantilever to maintain a stable oscillation amplitude is so high that the effect described above becomes irrelevant (Supporting Information File 3 contains experimental data of the instabilities when using conventional AM in vacuum).

**Figure 6:**
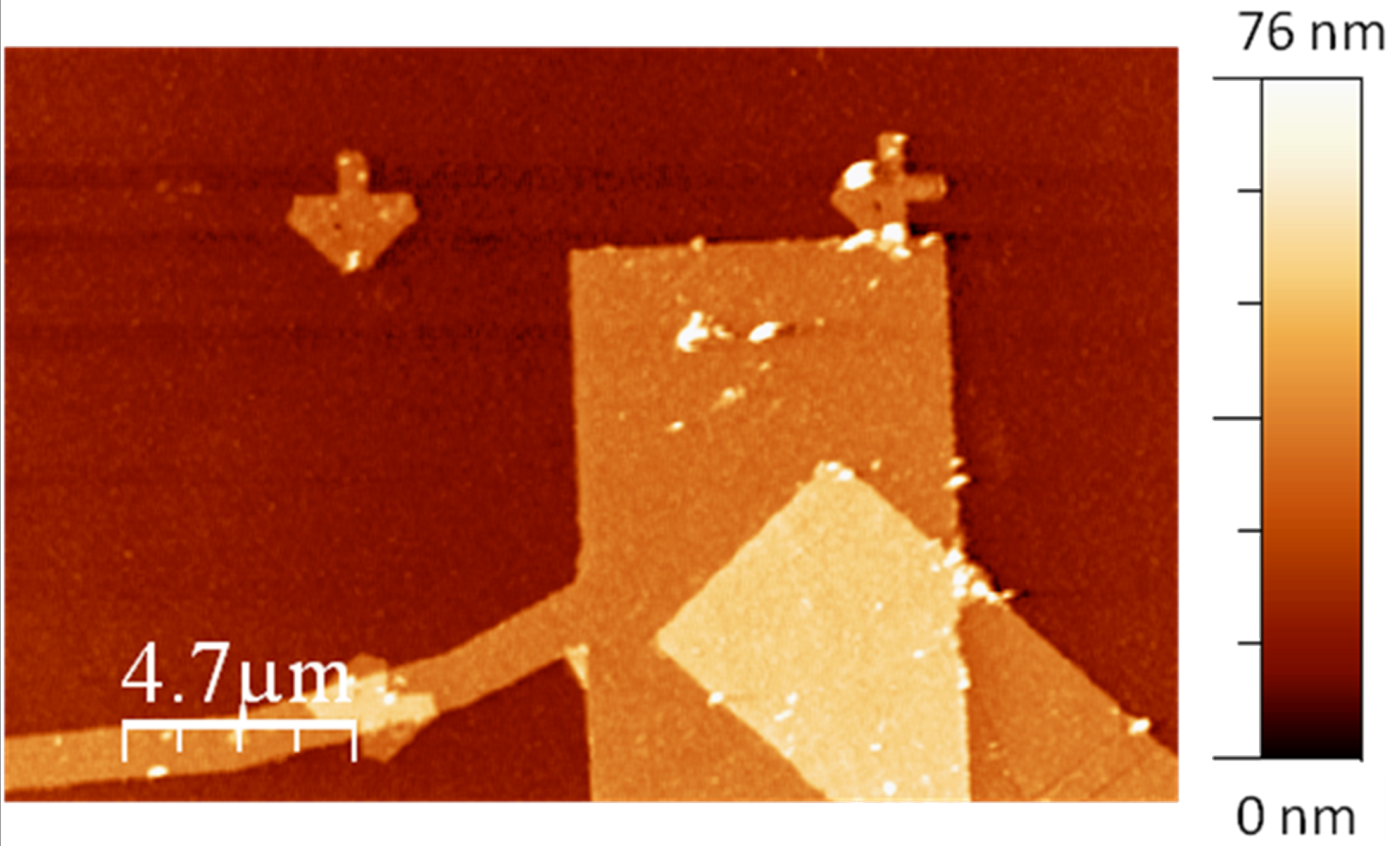
Gold electrodes fabricated by e-beam lithography. The DAM topography was acquired in vacuum with excellent stability despite the polymer contamination that is characteristic of the lithography process. Nanosensors PPP-FMR probe with: *A* = 24 nm; Q = 8600; *f*_0_ = 61.1 kHz; *k* = 1.3 N/m; line rate = 0.9 Hz; setpoint = 3.8 pW.

In addition to the grid sample we imaged a number of surfaces of technological and fundamental relevance using DAM (Supporting Information File 1 includes a variety of images taken in different environments). Figure 6 shows a silicon substrate on which several motives have been fabricated by means of a conventional e-beam lithography technique. The preparation of these samples involves several steps including deposition and lift-off of a polymer layer. This layer is, in many cases, very difficult to remove completely, leaving the sample contaminated. During scanning in FM in vacuum, the tip easily passes from the attractive to the repulsive regime, in which it is contaminated by the polymer.

### DAM-AFM in liquids

Low quality factors are common when imaging in liquids due to the viscous hydrodynamic loading between the cantilever and the environment. This friction in some cases induces an overdamped dynamic of the cantilever, making it very difficult to apply low forces in AM, which are necessary to obtain stable virus images [27], for example. Since the demonstration of true atomic resolution in liquids by Fukuma et al. [11] using FM [28], this mode has attracted the attention of the AFM community in attempts to image biological samples with high resolution. FM is able to overcome the limitations of AM making it possible to obtain high-quality images of the viruses and other biological samples [29–30]. However, FM is only stable while the tip is clean and the conservative interaction is repulsive, but once the tip becomes contaminated, which is very common when measuring biological samples under physiological conditions, the interaction curve is not monotonic, resulting in instabilities in the FM feedback.

**Figure 7:**
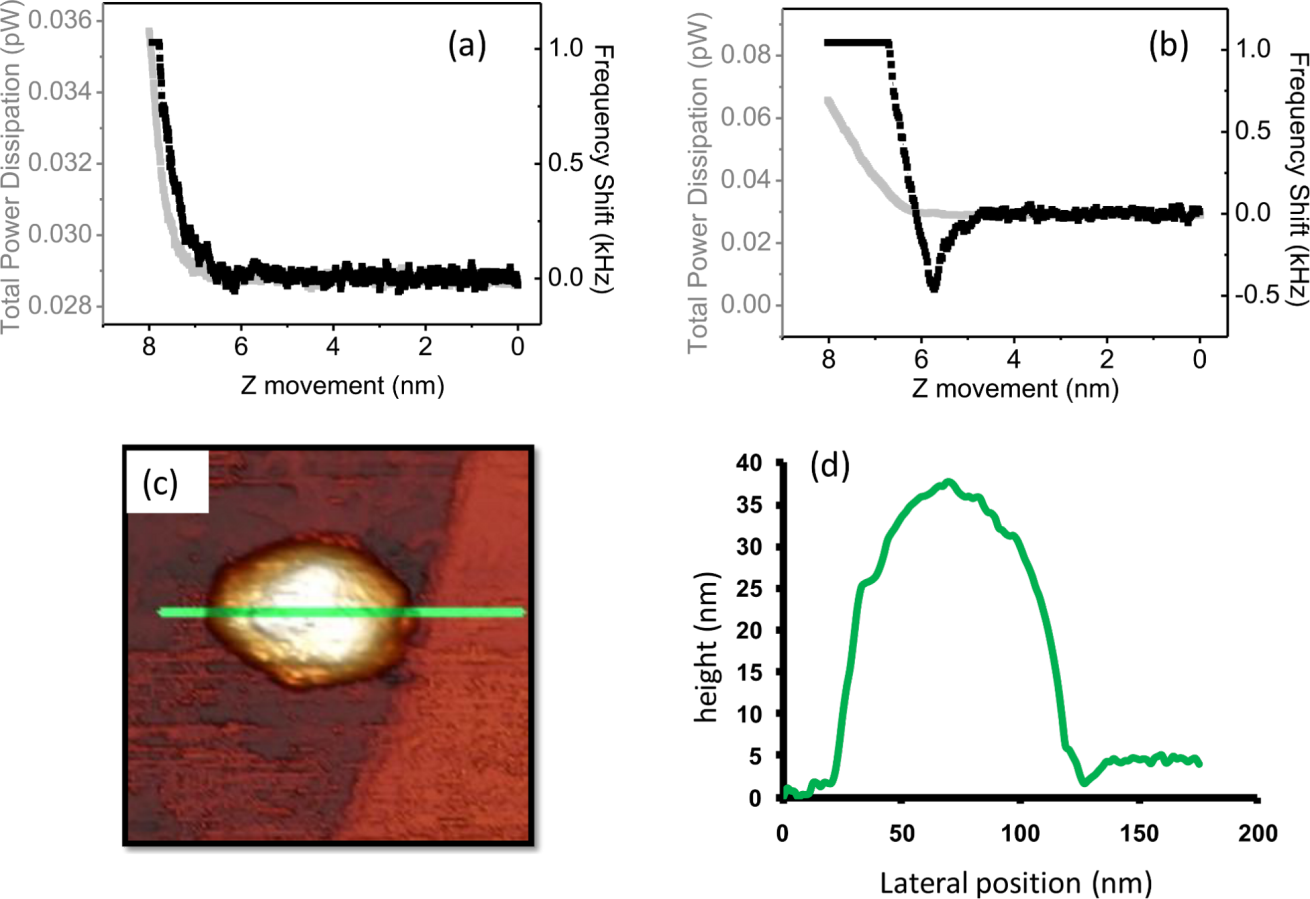
DAM in liquid. Frequency shift (black) and dissipation (light gray) for a clean tip (a) and after becoming contaminated (b). Note that the flat region of the frequency shift in (b) reflects the saturation of the PLL. (c) DAM topography showing a 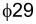 virus adsorbed on a HOPG substrate. (d) Height profile along the green line drawn in (c). Image parameters: *A* = 2 nm, *k* = 0.6 N/m, Q = 4, line rate = 2 Hz; *f*_0_ = 16 kHz, setpoint = 33 fW.

Figure 7a shows the dependence of the frequency shift and the dissipation for a clean AFM tip immersed in a buffer solution. Both magnitudes grow monotonically with the tip–sample distance. Figure 7b shows this dependence again with the same tip but this time contaminated after scanning a highly oriented pyrolytic graphite (HOPG) substrate with viruses adsorbed on it. While the dissipation is still monotonic, the frequency shift is not. This type of frequency-shift dependence makes scanning the surface impractical with FM. However, this is not an issue for DAM. Figure 7c displays an in-liquid DAM topography in which a 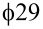 bacteriophage [31] adsorbed on a HOPG surface can be seen. Figure 7d shows a height profile along the green line drawn in Figure 7c. Notice that the virus topography exhibits the nominal height for 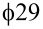 [32] implicating that the applied force is very low. By using Sader’s expression [33] the applied force can be calculated from the frequency-shift data. This value is nearly 100 pN, which is remarkable taking into account the relative high stiffness of the cantilever (0.6 N/m). In this case, DAM prevails over AM because the adhesion (attractive forces) on the virus is always lower than on the substrate, as can be easily verified by performing force versus distance curves [30]. Scanning in AM implies fixing a total energy for the cantilever that is high enough to enable scanning of the substrate without being trapped by the attractive forces, but this energy is also high enough to damage the virus. In DAM the energy is automatically adapted at each point of the image to optimize the image conditions.

## Conclusion

We have discussed the effects of the amplitude feedback on the transient times and energy balance, concluding that DAM is a suitable method for imaging in different environments ranging from vacuum to liquids and is useful for a variety of applications. DAM operation avoids the feedback instabilities associated with the transition between noncontact and intermittent-contact regimes. This feature translates to stable scanning of heterogeneous samples of technological relevance that are cumbersome to scan in vacuum, and which can be different to the standard samples used in UHV fundamental surface-science studies, e.g., atomically flat single crystals. Using DAM in liquids we have already been able to obtain true atomic resolution on a mica surface (see Supporting Information File 1) but atomic resolution in vacuum remains a challenge. DAM can also improve magnetic force imaging since it allows operating at smaller tip–sample distances than the conventional modes. Finally, since DAM reduces the settling time, it may be useful for high-speed scanning in air under ambient conditions.

## Supporting Information

File 1 DAM images of relevant technological samples.

File 2 Dynamic response in DAM-AFM.

File 3 Handling instabilities with AM and DAM.
