# Supplementary material for: Drive-amplitude-modulation atomic force microscopy: From vacuum to liquids
Source: Beilstein J Nanotechnol. 2012 Apr 18;3:336–44. doi: 10.3762/bjnano.3.38 (PMC3343270; doi:10.3762/bjnano.3.38)
Supplement: File 1 — DAM images of relevant technological samples. [file Beilstein_J_Nanotechnol-03-336-s001.pdf]

Supporting Information

for

Drive-amplitude-modulation atomic force microscopy: From vacuum to liquids

Miriam Jaafar[1], David Martínez-Martín*[1], Mariano Cuenca[2], John Melcher[3], Arvind Raman[4] and Julio Gómez-Herrero*[1]

Address: [1]Departamento de Física de la Materia Condensada, Universidad Autónoma de Madrid, E-28049 Madrid, Spain, [2]Servicios generales de apoyo a la investigación, Universidad Autónoma de Madrid, E-28049 Madrid, Spain, [3]Department of Engineering Mathematics, University of Bristol, Bristol BS8 1TR, United Kingdom and [4]Birck Nanotechnology Center and School of Mechanical Engineering, Purdue University, West Lafayette, IN 47904-2088, USA

Email: David Martínez-Martín - davidmmo@gmail.com; Julio Gomez-Herrero - julio.gomez@uam.es

* Corresponding author

DAM images of relevant technological samples

This document is designed to complete the information given in the manuscript and to show the performance of DAM on different technological surfaces, including a sample fabricated by e-beam lithography and a magnetic hard disk.

We begin by considering two topography images of the same calibration grid as used in the manuscript. Both topographies were acquired consecutively in DAM with or without PLL at the same scanning rate (1.4 Hz/line). The quality of both images is very similar, indicating that the narrow bandwidth (the Q was higher than 20000) is not a critical issue for DAM.

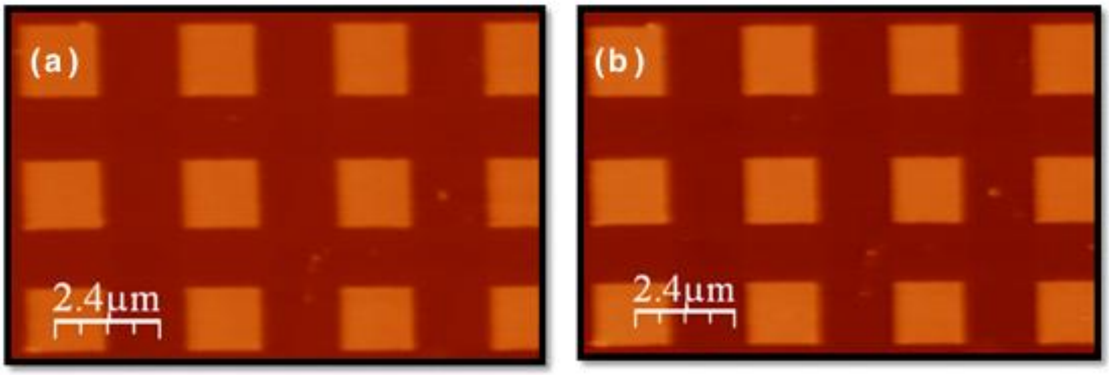

Figure S1: Shows two consecutive topographies acquired in DAM with (a) and without (b) PLL. Image parameters: A = 10 nm; k = 22 N/m; Q = 20000; line rate = 1.2 Hz; f_0 = 250 kHz; set point = 12 pW.

DAM can be easily used in air under ambient conditions. This application is trivial, and while we did not find, so far, any significant advantages with respect to AM we report for completeness a DAM topography image acquired in air of a gold film evaporated on a glass substrate.

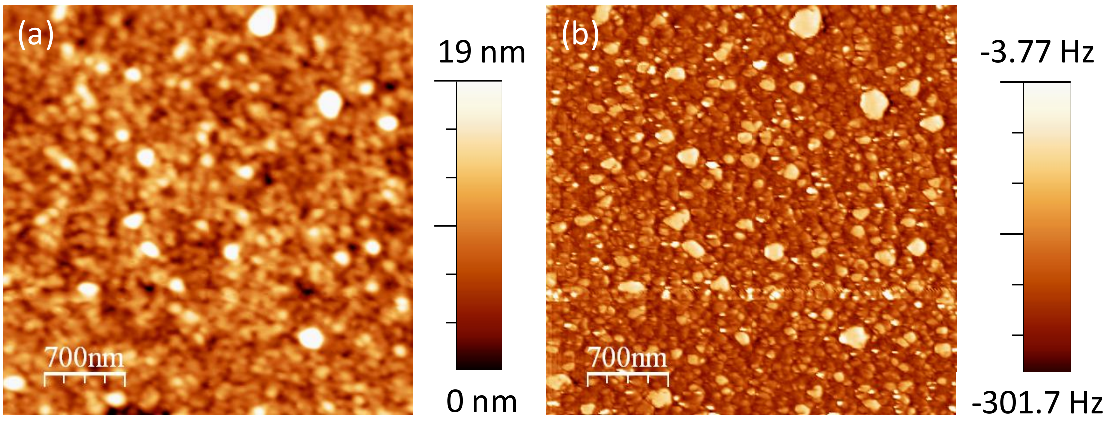

Figure S2: DAM topography (a) and frequency shift (b) images of a gold thin film deposited on a glass substrate, acquired under ambient conditions. Image parameters: A = 12 nm; k = 15 N/m; Q = 450; line rate= 1.48 Hz; f_0 = 225 kHz; set point = 4.6 pW.

Additionally we report magnetic force microscopy (MFM) images using DAM in combination with a PLL. Figure S3 shows an in-vacuum DAM topography, and the corresponding frequency shift image taken in lift mode, of a high-density hard disk. We recall that FM-AFM was originally introduced as a method to increase the sensitivity in MFM measurements.

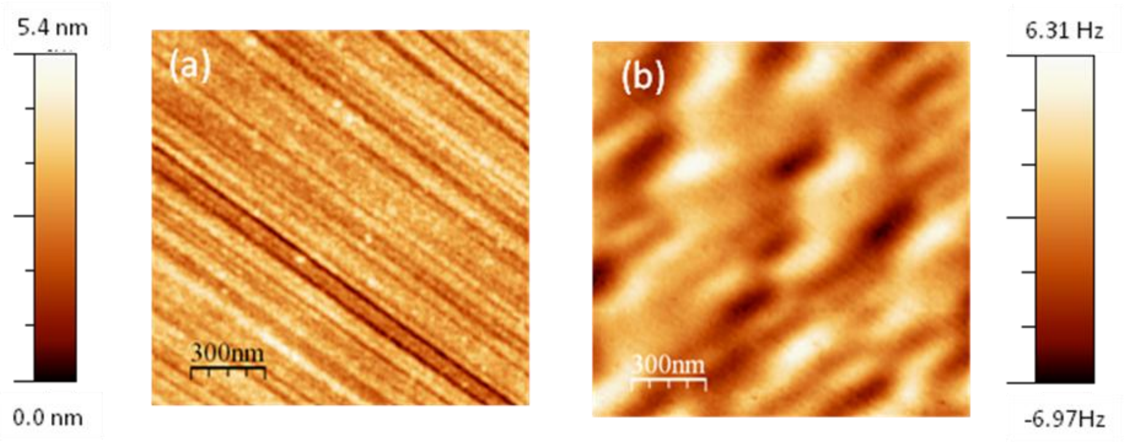

Figure S3: High-density hard disk. (a) DAM topography image. (b) Frequency shift image obtained at a lift-mode distance of 35 nm showing the magnetic interaction between a Nanosensors PPP-MFMR probe and the surface. The topography image is free from the influence of long-range forces as is the case of the magnetic interaction. Image parameters: A = 10 nm; Q = 8000; f_0 = 72.9 kHz; k = 1.6 N/m; Set Point = 0.7 pW.

Regarding the quality of the images obtained with DAM, this method is capable of reaching true atomic resolution while working in liquids. Figure S4 shows a true atomic-resolution image of a mica surface immersed in liquid.

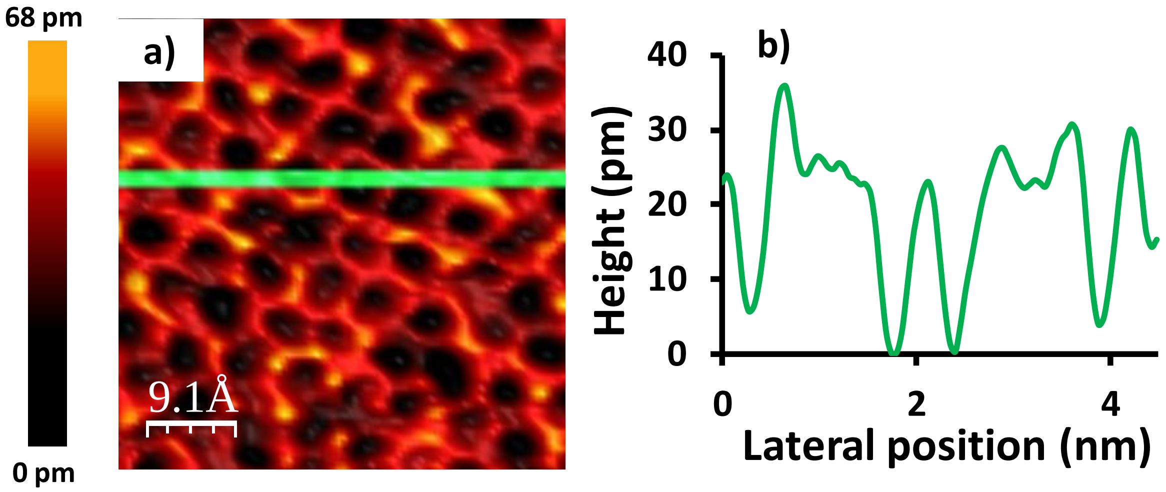

Figure S4: True atomic resolution in liquids with DAM. (a) DAM topography (render 3D) of a mica surface immersed in pure water. The image shows true atomic resolution. (b) is the corresponding profile across image (a). Image parameters: A = 0.7 nm, Q = 7; f_0 = 141 kHz; k = 26 N/m; line rate = 11 Hz;
