# Supplementary material for: Drive-amplitude-modulation atomic force microscopy: From vacuum to liquids
Source: Beilstein J Nanotechnol. 2012 Apr 18;3:336–44. doi: 10.3762/bjnano.3.38 (PMC3343270; doi:10.3762/bjnano.3.38)

Supporting Information

for

Drive-amplitude-modulation atomic force microscopy: From vacuum to liquids

Miriam Jaafar[1], David Martínez-Martín*[1], Mariano Cuenca[2], John Melcher[3], Arvind Raman[4] and Julio Gómez-Herrero*[1]

Address: [1]Departamento de Física de la Materia Condensada, Universidad Autónoma de Madrid, E-28049 Madrid, Spain, [2]Servicios generales de apoyo a la investigación, Universidad Autónoma de Madrid, E-28049 Madrid, Spain, [3]Department of Engineering Mathematics, University of Bristol, Bristol BS8 1TR, United Kingdom and [4]Birck Nanotechnology Center and School of Mechanical Engineering, Purdue University, West Lafayette, IN 47904-2088, USA

Email: David Martínez-Martín - davidmmo@gmail.com; Julio Gomez-Herrero - julio.gomez@uam.es

* Corresponding author

Dynamic response in DAM-AFM

Within the point-mass approximation a cantilever can be seen as a mass on a spring. Therefore the equation of motion is given by:

$$\frac{d^2x}{dt^2} + \frac{\omega_0}{q}\frac{dx}{dt} + \omega_0^2 = A\cos\left(\omega_f t\right)$$

The general solution can be written as:

$$x(t) = \frac{A}{({\omega_0}^2 - {\omega_f}^2) + (\frac{\omega_0}{q}\omega_f)^2}\left(\left({\omega_0}^2 - {\omega_f}^2\right)\left(\cos\left(\omega_f t\right) - e^{-\frac{\omega_0}{2q}t} cos(\omega_0 t)\right) + \frac{\omega_0}{q}\omega_f\left(\sin(\omega_0 t) - \frac{{\omega_0}^2 + {\omega_f}^2}{2\omega_0\omega_f} e^{-\frac{\omega_0}{2q}t} sin(\omega_0 t)\right)\right)$$

The use of a phase-locked loop implies that $\omega_0 = \omega_f$ and thus:

$$x(t) = \frac{Aq}{{\omega_0}^2}\left(\sin(\omega_0 t) - e^{-\frac{\omega_0}{2q}t} sin(\omega_0 t)\right)$$

The standard procedure to readout the oscillation amplitude is to use a lock-in amplifier, which first multiplies the previous solution by a reference signal $(\omega_0 t)$. The result of this operation is a dc component proportional to the signal amplitude plus a $2\omega_0$ component, which is usually removed with a low-pass filter. After these operations the output of the lock-in amplifier reads as:

$$A_{out} = \frac{Aq}{2{\omega_0}^2}\left(1 - e^{-\frac{\omega_0}{2q}t}\right)$$

With a constant time $\tau = \frac{2q}{\omega_0}$

This corresponds to a system with a transfer function G:

$$G = \frac{\frac{1}{4\omega_0}}{s + \frac{\omega_0}{2q}}$$

$$H = K_p$$

If feedback is performed then these values are modified as follows:

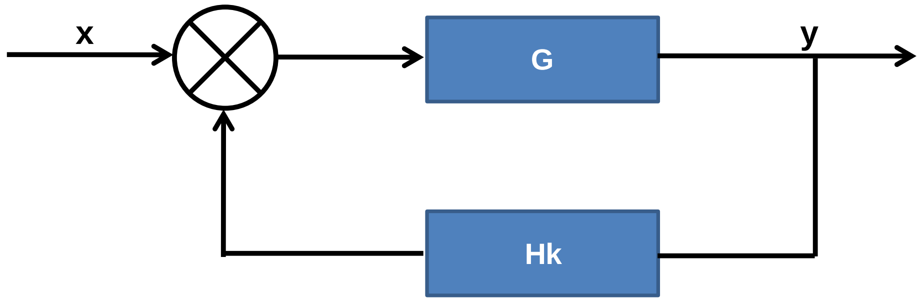

$$\frac{y}{x} = \frac{G}{1 + GK}$$

$$\frac{y}{x} = \frac{\frac{1}{4\omega_0}}{s + \frac{\omega_0}{2q} + \frac{K_p}{4\omega_0}}$$

And the time response:

$$A_{out} = \frac{Aq}{2\omega_0{}^2 + K_p q}\left(1 - e^{-\left(\frac{\omega_0}{2q} + \frac{K_p}{4\omega_0}\right)t}\right)$$

As we can see, the new time constant is:

$$\tau = \frac{4q\omega_0}{2\omega_0{}^2 + K_p q}$$

This involves the gain K_p of the feedback loop. As a consequence the time response can be arbitrarily reduced by tuning the feedback gain.

This analysis was performed using a feedback that only includes a proportional gain, since this is the most important parameter to regulate the amplitude of cantilever. However the analysis could be easily generalized to include an integral or even a differential gain.

Open loop

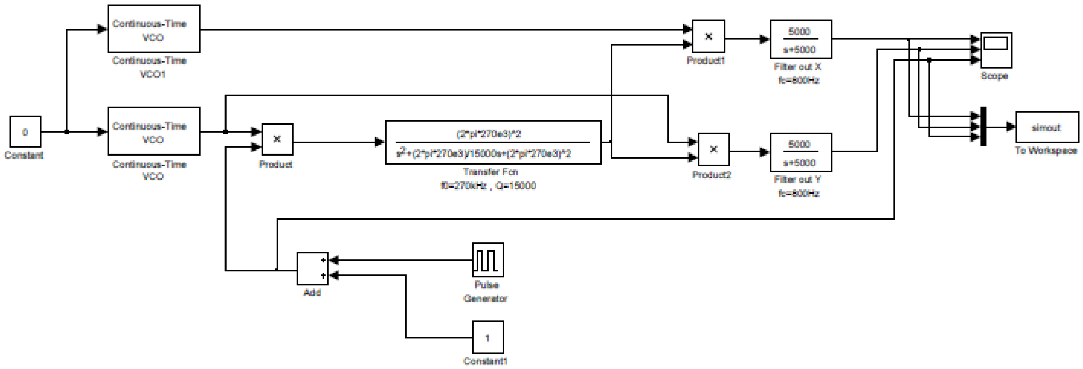

Closed loop

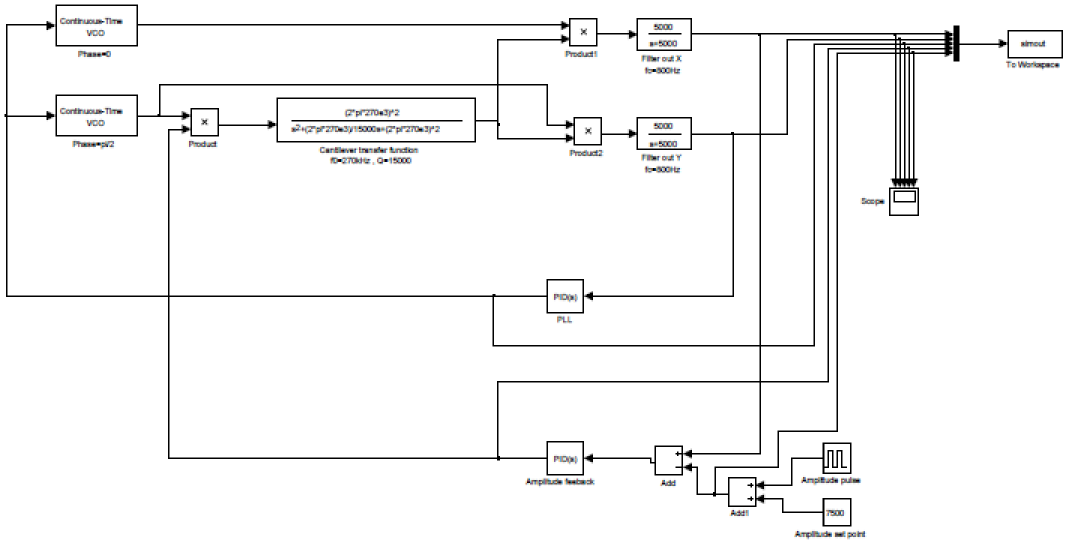

Supplement: File 2 — Dynamic response in DAM-AFM. [file Beilstein_J_Nanotechnol-03-336-s002.pdf]
