# Supplementary material for: Drive-amplitude-modulation atomic force microscopy: From vacuum to liquids
Source: Beilstein J Nanotechnol. 2012 Apr 18;3:336–44. doi: 10.3762/bjnano.3.38 (PMC3343270; doi:10.3762/bjnano.3.38)
Supplement: File 3 — Handling instabilities with AM and DAM. [file Beilstein_J_Nanotechnol-03-336-s003.pdf]

Supporting Information

for

Drive-amplitude-modulation atomic force microscopy: From vacuum to liquids

Miriam Jaafar[1], David Martínez-Martín*[1], Mariano Cuenca[2], John Melcher[3], Arvind Raman[4] and Julio Gómez-Herrero*[1]

Address: [1]Departamento de Física de la Materia Condensada, Universidad Autónoma de Madrid, E-28049 Madrid, Spain, [2]Servicios generales de apoyo a la investigación, Universidad Autónoma de Madrid, E-28049 Madrid, Spain, [3]Department of Engineering Mathematics, University of Bristol, Bristol BS8 1TR, United Kingdom and [4]Birck Nanotechnology Center and School of Mechanical Engineering, Purdue University, West Lafayette, IN 47904-2088, USA

Email: David Martínez-Martín - davidmmo@gmail.com; Julio Gomez-Herrero - julio.gomez@uam.es

* Corresponding author

Handling instabilities with AM and DAM

Figure S5 shows a set of oscilloscope screen captures of the oscillation amplitude as a function of time in which we used an AM setup with a PLL for a tip with a clear bistable behavior. The tip position is fixed and the cantilever oscillation amplitude is about 15 nm. The differences between the images are small variations in the cantilever driving amplitude or setpoint. According to the previous lines, the energy required by the cantilever is barely enough to keep the system at resonance and any loss originating from tip–sample interaction results in a tip crash (the oscillation amplitude vanishes). The system jumped from state (d) to state (e) without any apparent variation of the experimental conditions (small variations of the tip position originating from thermal drift are to be expected). Finally, a small tap with a finger on the microscope chamber wall moved the system state from (e) to (f). The obvious interpretation is that there are several possible trajectories with similar energies in phase space.

Following F. J. Giessibl [1] we can summarize this discussion in the conjecture

$$\pi \cdot k\, a^{2} \geq e_{ts}\, Q$$

For the sake of comparison in AM, assuming similar tip–sample dissipation in both *vacuum* and *air*, stable scanning *in vacuum* (high Q) requires much larger amplitude than *in air* (low Q).

AM can be still used in vacuum for soft topographies but is useless for demanding samples, and this is not only because of the long settling time or the narrow-bandwidth problems but also because of the dissipation problem. It can

be argued that by increasing the amplitude by a factor 4–5 one can recover the stability, but the price to be paid is a substantial drop in the sensitivity, high nonlinearities and possible sample damage at the atomic scale.

For DAM the situation is quite different; we are pumping energy with feedback, so if the amplitude drops, the driving force becomes higher increasing the energy of the cantilever, and thus keeping the amplitude constant and avoiding tip crash. Figure S5g,h display the amplitude and dissipation as a function of time for the same conditions used in Figure S5a–f. In this case, we use the DAM feedback to maintain a constant amplitude.

For high enough Q and/or low enough amplitude, FM and DAM can still present stability problems related to the energy balance because small changes in e_{ts} require a major correction in the driving force [1]. Nevertheless since DAM does not present problems in the noncontact-to-contact transition its stability is higher.

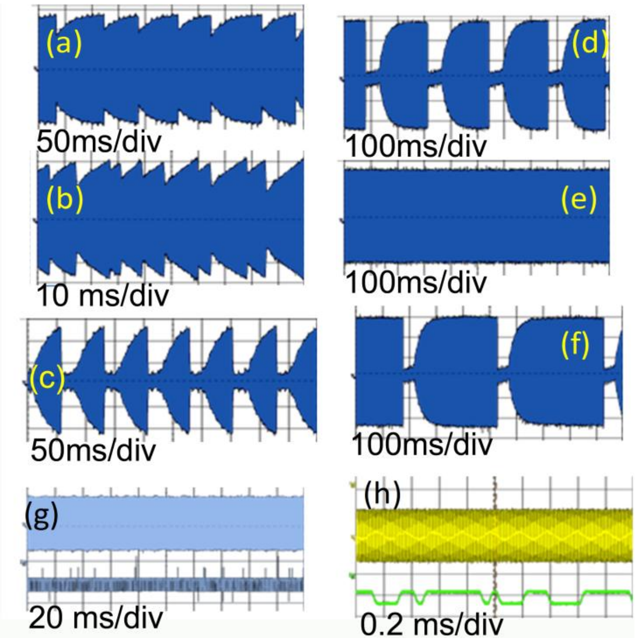

Figure S5: Oscilloscope-captured images of the cantilever oscillation amplitude as a function of time taken in AM (a–f) and DAM (g,h). In AM small amplitude variations (a,b) or setpoint variations (b–d), spontaneous events (d–e) or a soft mechanical perturbation on the vacuum chamber wall result in a variety of instabilities. DAM keeps the amplitude constant by pumping energy into the cantilever. *K* = 23 N/m, Q = 16475, amplitude ~10 nm.

References

1. Giessibl, F. J. *Rev. Mod. Phys.* **2003,** *75,* 949–983.

doi:10.1103/RevModPhys.75.949
